# Attempting to Increase the Effectiveness of the Antidepressant Trazodone Hydrochloride Drug Using π-Acceptors

**DOI:** 10.3390/ijerph191811281

**Published:** 2022-09-08

**Authors:** Walaa F. Alsanie, Majid Alhomrani, Abdulhakeem S. Alamri, Hussain Alyami, Sonam Shakya, Hamza Habeeballah, Heba A. Alkhatabi, Raed I. Felimban, Abdulwahab Alamri, Abdulhameed Abdullah Alhabeeb, Bassem M. Raafat, Moamen S. Refat, Ahmed Gaber

**Affiliations:** 1 Department of Clinical Laboratories Sciences, The Faculty of Applied Medical Sciences, Taif University, Taif 21944, Saudi Arabia; 2 Centre of Biomedical Sciences Research (CBSR), Deanship of Scientific Research, Taif University, Taif 21944, Saudi Arabia; 3 College of Medicine, Taif University, Taif 21944, Saudi Arabia; 4 Department of Chemistry, Faculty of Science, Aligarh Muslim University, Aligarh 202002, India; 5 Department of Medical Laboratory Technology, Faculty of Applied Medical Sciences in Rabigh, King Abdulaziz University, Jeddah 21589, Saudi Arabia; 6 Department of Medical Laboratory Technology, Faculty of Applied Medical Sciences, King Abdulaziz University, Jeddah 21589, Saudi Arabia; 7 Center of Excellence in Genomic Medicine Research (CEGMR), King Abdulaziz University, Jeddah 21589, Saudi Arabia; 8 King Fahd Medical Research Centre, Hematology Research Unit, King Abdulaziz University, Jeddah 21589, Saudi Arabia; 9 Center of Innovation in Personalized Medicine (CIPM), 3D Bioprinting Unit, King Abdulaziz University, Jeddah 21589, Saudi Arabia; 10 Department of Pharmacology and Toxicology, College of Pharmacy, University of Hail, Hail 81442, Saudi Arabia; 11 National Centre for Mental Health Promotion, Riyadh 11525, Saudi Arabia; 12 Department of Radiological Sciences, College of Applied Medical Sciences, Taif University, Taif 21944, Saudi Arabia; 13 Department of Chemistry, College of Science, Taif University, Taif 21944, Saudi Arabia; 14 Department of Biology, College of Science, Taif University, Taif 21944, Saudi Arabia

**Keywords:** trazodone HCl, depression, charge transfer complex, spectroscopic, molecular docking

## Abstract

Major depressive disorder is a prevalent mood illness that is mildly heritable. Cases with the highest familial risk had recurrence and onset at a young age. Trazodone hydrochloride is an antidepressant medicine that affects the chemical messengers in the brain known as neurotransmitters, which include acetylcholine, norepinephrine, dopamine, and serotonin. In the present research, in solid and liquid phases, the 1:1 charge-transfer complexes between trazodone hydrochloride (TZD) and six different π-acceptors were synthesized and investigated using different microscopic techniques. The relation of dative ion pairs [TZD+, A−], where A is the acceptor, was inferred via intermolecular charge-transfer complexes. Additionally, a molecular docking examination was utilized to compare the interactions of protein receptors (serotonin-6BQH) with the TZD alone or in combination with the six distinct acceptor charge-transfer complexes. To refine the docking results acquired from AutoDock Vina and to better examine the molecular mechanisms of receptor-ligand interactions, a 100 ns run of molecular dynamics simulation was used. All the results obtained in this study prove that the 2,6-dichloroquinone-4-chloroimide (DCQ)/TZD complex interacts with serotonin receptors more efficiently than reactant donor TZD only and that [(TZD)(DCQ)]-serotonin has the highest binding energy value of all π-acceptor complexes.

## 1. Introduction

Major depressive disorder (MDD) or clinical depression creates severe symptoms that interfere with the person’s ability to sleep, eat, and work [1]. It is the world’s third greatest cause of years spent disabled and a key factor in early suicide mortality [2]. It is a disease that can strike anyone of any age, race, income, culture, or academic level. Depression is thought to be caused by genetic, biochemical, environmental, and psychological factors [3,4,5].

The third most often used therapeutic drug class worldwide is antidepressants [6]. Most of these drugs work by using substances that affect the serotonin transporter, a single protein in the brain (5-HT: a group of G protein-coupled receptors). Approximately 80% of all currently marketed antidepressant drugs are selective serotonin reuptake inhibitors (SSRIs), which stop 5-HT reuptake [7]. Both 5-HT(2A) and 5-HT(2C) receptors are G-protein-coupled receptors (GPCRs), which constitute a superfamily of receptors. G-proteins and GPCRs work together to carry extracellular signals inside cells. Numerous medications have an impact on anxiety, insomnia, depression, hallucinations, schizophrenia, dysthymia, eating patterns, and neuro-endocrine functions are mediated via the 5-HT(2A) and 5-HT(2C) receptors.

Trazodone hydrochloride (TZD) was the first triazolopyridine derivative to be created for the treatment of serious depression, therefore, it was the second-generation antidepressant drug [8]. Thus, TZD is used to treat depression and has been demonstrated to be effective in lowering the majority of symptoms associated with depression [9,10,11]. Medicines with many therapeutic mechanisms are referred to as multifunctional drugs. TZD is a multipurpose medication having pharmacologic effects that depend on the dose [11]. TZD’s unique multifunctional pharmacological profile explains its effectiveness in treating MDD [11,12]. It has hypnotic effects at low levels because it blocks 5-HT2A receptors, H1 histamine receptors, and α1 adrenergic receptors. When used in higher doses, TZD becomes an antidepressant by enlisting the blockage of the serotonin transporter (SERT) [11,12]. TZD differs from traditional antidepressants (MAO-I) pharmacologically since it has no effect on monoamine oxidase (MAO) activity [13] and has only minor effects on norepinephrine reuptake [14]. It is well known that depression is caused by an imbalance of various neurotransmitters. TZD is an antidepressant that modifies the neurotransmitters that the brain’s nerves use to communicate with one another. These neurotransmitters include acetylcholine, norepinephrine, dopamine, and serotonin. Although trazodone’s exact mode of action is uncertain, it is likely that it lessens depressive symptoms by preventing brain nerves from absorbing serotonin. Therefore, more serotonin is produced as a result, stimulating additional nerves [8,14]

When two molecules contact, a portion of the electronic charge is transferred between them. This process is known as a charge-transfer (CT) complex, also referred to as an electron-donor-acceptor complex. The electrostatic attraction that results stabilizes the molecular complex. The molecule from which the charge is transferred is the source, and the molecule receiving it is the electron acceptor. The CT electron donor interaction is also significant in drug-receptor binding mechanisms [15] and in a variety of biological disciplines [16]. CT interactions of some acceptors, on the other hand, have been successfully exploited in a pharmacokinetic study [17]. CT-receptor drugs have been extensively studied for these broad applications [18]. An “π-acceptor ligand” is a ligand that can donate a pair of electrons from a lone pair to the metal center while accepting electron density from the metal’s δ orbitals into either δ orbitals or π-antibonding orbitals. While a ligand known as a σ-donor donates electrons from a lone pair to the metal center, acting as a Lewis base. Several reports documented the utilization and benefit of π-acceptors in the spectrophotometric purpose of various medicines in pharmaceutical formulations [19,20,21,22,23,24,25].

In the present study, the 1:1 charge-transfer complexes between TZD and six distinct π-acceptors in solid and liquid phases were synthesized and investigated using different microscopic techniques. Moreover, the molecular docking technique using Autodock Vina software was used for studying the interactions between TZD ligand alone or TZD along with the six CT complexes against serotonin (5-HT2C) receptor.

## 2. Materials and Methods

### 2.1. Preface

**Figure 1:**
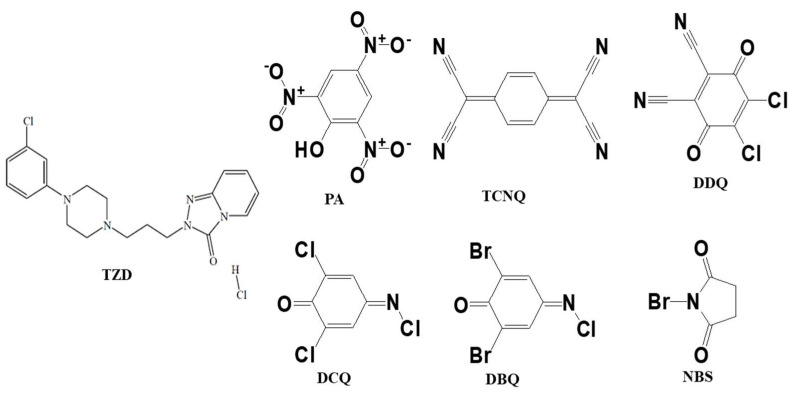
Structures of TZD and π-acceptors.

The only top-quality chemicals and reagents were obtained from Sigma and Fluka. The following substances were used without further purification: TZD, picric acid (PA), 2,3-dichloro-5,6-dicyano-p-benzoquinone (DDQ), tetracyanoquinodimethane (TCNQ), 2,6-dichloroquinone-4-chloroimide (DCQ), 2,6-dibromoquinone-4-chloroimide (DBQ), and N-bromosuccinimide (NBS). The structures of TZD and π-acceptors are shown in Figure 1.

The six solid powder complexes were synthesized by combining (1 mmol, 0.410 g) of TZD hydrochloride in 1 mmol of each acceptor [26].

### 2.2. Molecular Docking

OpenBabelIGUI software version 2.4.1(http://openbabel.org, accessed on 1 February 2022) was used to obtain the structure of TZD with the six CT complexes in PDBQT format [27].

MMFF94 force field and conjugate gradient optimization algorithm were used to minimize the energy of the structures through PyRx-Python prescription 0.8 for 500 steps [28]. The RCSB protein data library provided the 3D crystal structures of serotonin (PDB ID: 6BQH) and dopamine (PDB ID: 6CM4) [29]. Using BIOVIA Discovery Studio Visualizer version 19 (Waltham, MA, USA), the receptors were prepped for docking by eliminating the native ligand and additional heteroatoms, including water. Autodock Tool was used to add polar hydrogen atoms and determine the Kollman charges of the receptors [30]. Geistenger technique was employed to assign partial charges. The docking calculations were done with Autodock Vina [31]. DS Visualizer (Available online: https://www.3ds.com/products-services/biovia/ (accessed on 1 February 2022) was used to check the interactions of the docked positions.

### 2.3. Molecular Dynamics (MD) Simulation Study

Using the GROMACS program, the [(TZD)(DCQ)] complex and TZD alone were applied with the highest docking score for the MD simulation. The most recent versions of CGenFF and CHARMM-GUI were used to retrieve the parameter files and topology of the ligand [32].

**Figure 2:**
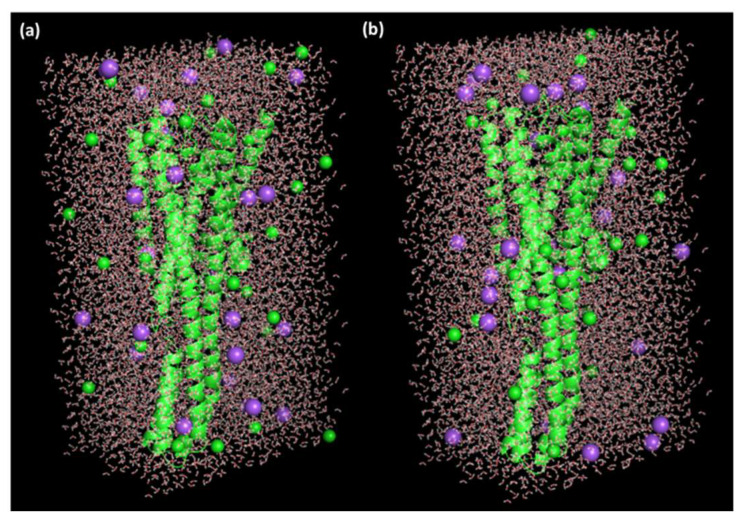
A triclinic box of (**a**) TZD drug alone and (**b**) [(TZD)(DCQ)].

The SPC water models were employed for explaining receptor-ligand structures [33]. 0.15 M salt (28 Na^+^ and 29 Cl^−^ ions) were added to neutralize the systems and mimic physiological salt concentrations (Figure 2).

In the NPT/NVT equilibration run, both systems were subjected to periodic boundary requirements using a Leap-frog MD integrator at constant temperatures of 300 K and pressures of 1.0 bar for a maximum time of 100 ns [34]. Energy reduction utilizing the steepest descent approach with 5000 steps was carried out [35] to reduce poor contact inside the system. Trajectory analysis was carried out using GROMACS analytical methods [36]. The root mean square deviation (RMSD) was calculated using the gmx rms tools and hydrogen bondings were investigated using the gmx hbond tool. The plots were completed by Grace Software [37,38].

### 2.4. Density Functional Theory

Density function theory was performed using the Gaussian 09RevD.01 package [39]. A stable molecular geometry was obtained to theoretically investigate the electronic transitions in the charge transfer complex [(TZD)(TCNQ)]. Pople’s basic set B3LYP/6-311G++ [40] was used, along with gradient corrected correlation to acquire the optimized structure of the CT complex [(TZD)(TCNQ)]. The CT complex’s electrostatic potential map (MEP), lowest unoccupied molecular orbital (LUMO), and highest occupied molecular orbital (HOMO) were also investigated [41]. For visualization, ChemCraft 1.5 software was used [42].

## 3. Results and Discussion

### 3.1. Multi-Spectroscopic Investigation

**Figure 3:**
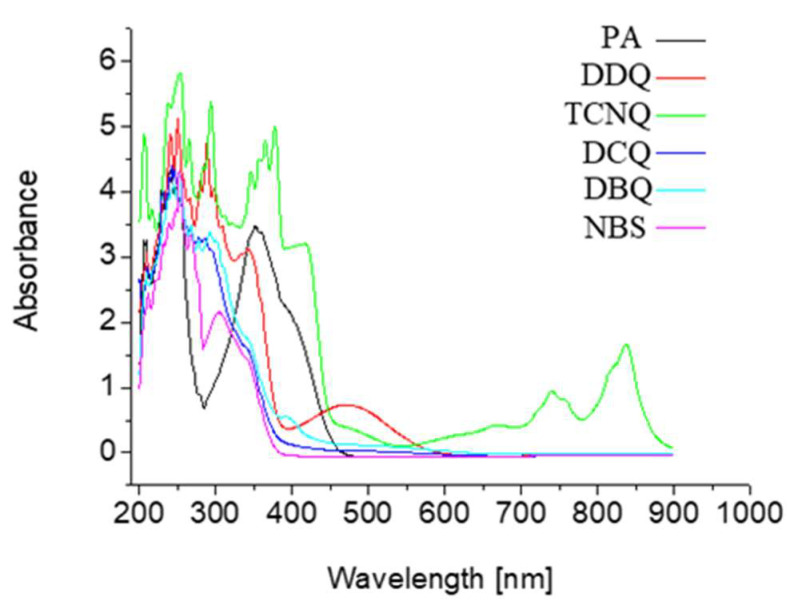
UV-Vis spectra of the TZD charge transfer complexes with the six π-acceptors.

UV-Vis spectra of the charge transfer systems of the TZD as a donor with six π-acceptors were run and exhibited in Figure 3. Elements analyze the carbon, hydrogen, and nitrogen contents’ data for the colored TZD charge transfer complexes in good agreement with the optical titration ratios between TZD and π-receptors.

**Figure 4:**
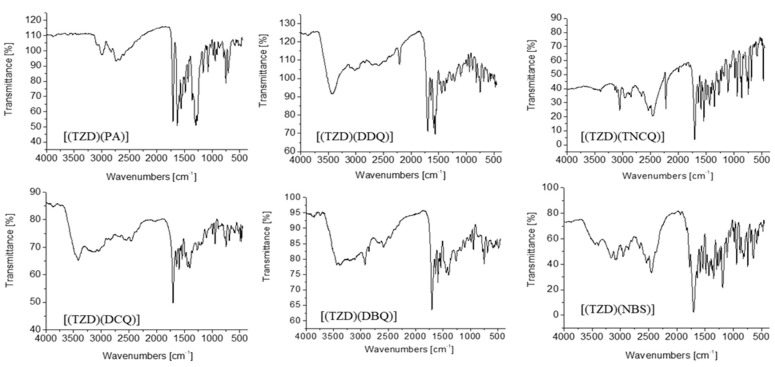
IR spectra of the ligand-acceptors complexes.

In the infrared spectra of free TZD, there are distinctive bands at 3000, 2954, 1704, 1650, 1600, 1350, and 750 cm^−1^, which are vibrating bands extended to C-_Haromatic_, C-H_aliphatic_, C=O, C=N, and C=C, C–N, and C–Cl, respectively. The IR spectra of the TZD drug with six π-acceptors are shown in Figure 4.

**Figure 5:**
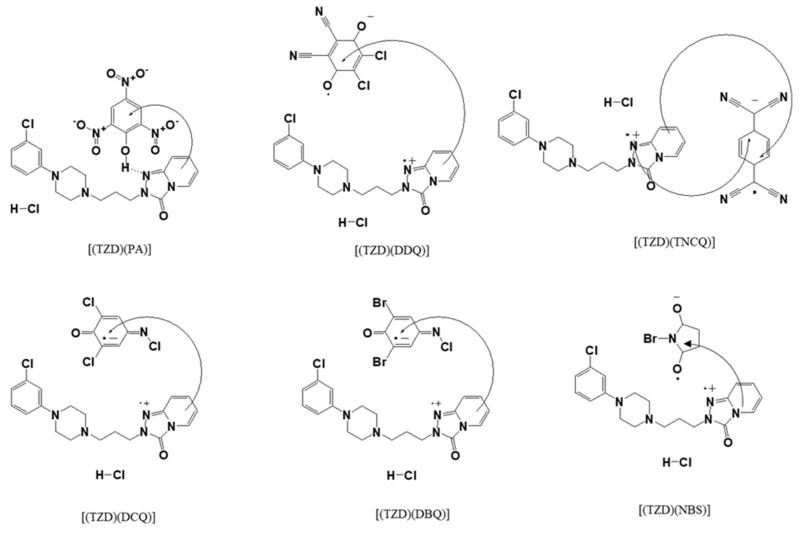
Speculated structures of the TZD-CT complexes.

IR spectra of the ligand-acceptor complexes revealed a combination of basal bands typical of both the TZD donor and an individual acceptor (Figure 4). The connection of intermolecular charge transfer complexes between the donor and acceptor was confirmed and supported by these findings. Small shifts in the donor and acceptor band intensities and wave values in TZD complexes rather than the molecules of the free reactants are due to the expected changes in the reactants’ molecular symmetries and electronic structures upon complexing. The v(C=N) and (C=C) vibrations of TZD alone are seen at 1650 and 1600 cm^−1^, respectively, however, these bands are displaced to lower wave numbers at 1627 and 1553 cm^−1^ in the case of the PA charge transfer complex. This is supported by the fact that the n–π* and π–π* molecular charge transfer complexes are located as indicated in Figure 4 and Figure 5.

Although the v(C=N) and (C=C) vibrations of TZD alone are visible at 1650 and 1600 cm^−1^, respectively, the PA charge transfer complex causes these bands to shift to lower wave numbers at 1627 and 1553 cm^−1^. The location of the n–π* and π–π* molecular charge transfer complexes, as shown in Figure 4 and Figure 5, supports this.

The (CN) vibrations of DDQ were at 2223 cm^−1^ and for TCNQ at 2234 cm^−1^ when they have been alone. These vibrations take place at 2213 cm^−1^ for DDQ and 2228 cm^−1^ for TCNQ, following complexing with TZD-DDQ and TZD-TCNQ. The v(C=N) and (C=C) vibrations in the case of TZD exhibit additional changes, shifting to (1643 and 1560) cm^−1^ and (1636 and 1537) cm^−1^, respectively. These results approved the formulation of the structures of the TZD-DDQ and TZD-TCNQ charge transfer complexes (Figure 4 and Figure 5). The ν(C=N) and ν(C=C) vibrations, on the other hand, were converted from 1650 to (1636–1643) cm^−1^ and 1600 to 1590 cm^−1^ in a row for the TZD-DCQ, TZD-DBQ, and TZD-NBS systems after complexing. It is confirmed by these variations in wavenumber values with increasing complexity that the acceptors’ empty π* orbitals should receive the electron donation from the TZD [43]. The D_HOMO_→D_LUMO_ transition is reflected by the shift in the IR bands of the acceptor and donor portions to lower wave numbers and higher values of the donor portion, respectively, to accept charge transfer for the n–π* interaction [44].

**Figure 6:**
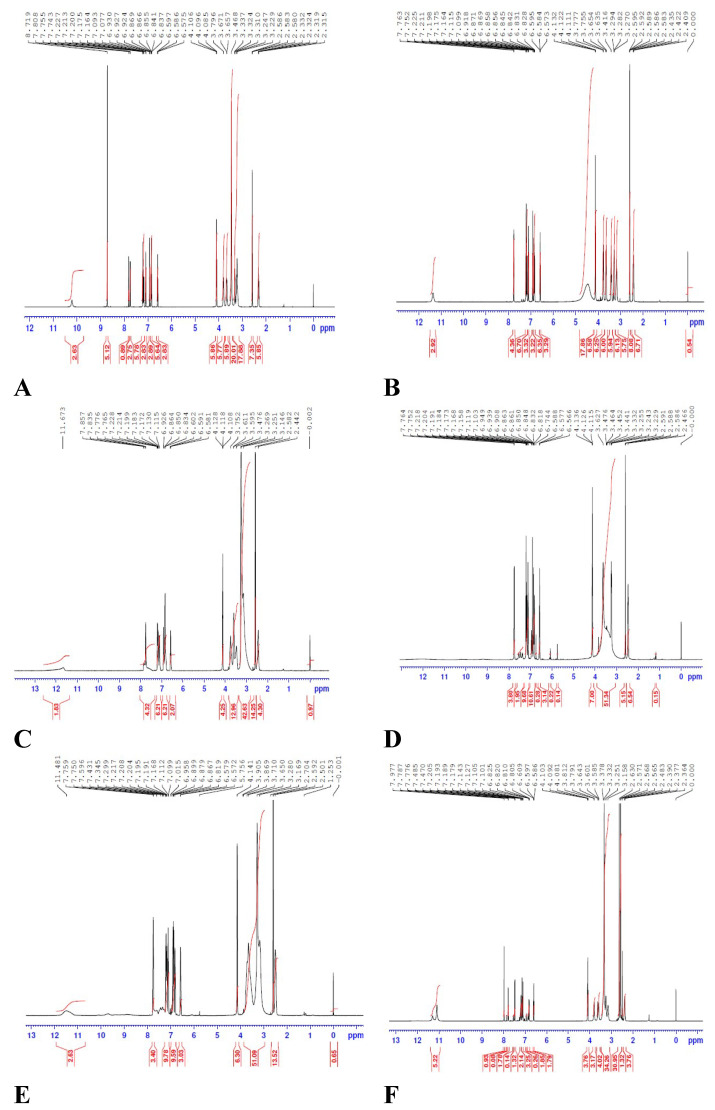
^1^HNMR spectra of the TZD drug with the six π-acceptors; (**A**): PA, (**B**): DDQ, (**C**): TCNQ, (**D**): DCQ, (**E**): DBQ, and (**F**): NBS.

Figure 6 illustrates the ^1^HNMR spectra of the TZD with the six π-acceptors complexes. The peaks of the aromatic rings, as well as the methylene protons, have been displaced to the lower field, indicating that CT complexes have been established. CT transitions take place during the complexing process when an electron is excited from the donor’s HOMO to the acceptor’s LUMO. The lowest charge transfer transition would include the amplification of an electron from the donor to the acceptor in the high occupancy molecular orbital (HOMO).

TZD and π-receptor interactions result in n-π* transitions and form the pairs of radical ions (the D^+.^ radical cation and the A^−^ radical anions). In the presence of the six receptors, conductivity values for TZD were measured, and a modest increase was observed. This small increase in conductivity after complexing supports the hypothesis that the charge transfer complex is made up of dative ion pairs [(TZD^+^) (acceptor^−^)].

**Figure 7:**
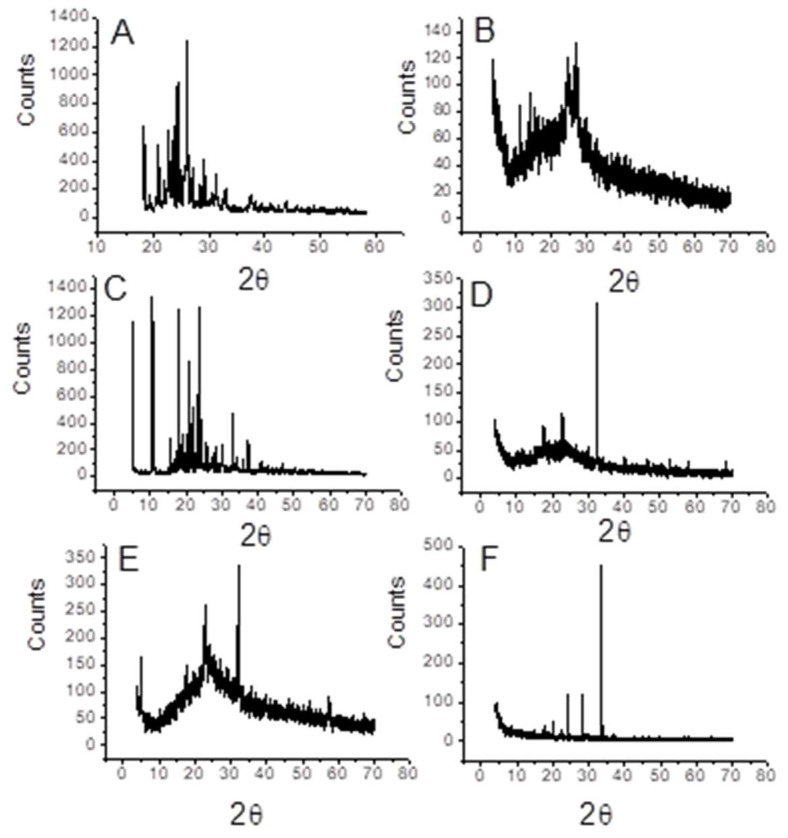
X-ray diffraction scans of the TZD with the six π-acceptors; (**A**): PA, (**B**): DDQ, (**C**): TCNQ, (**D**): DCQ, (**E**): DBQ, and (**F**): NBS.

**Figure 8:**
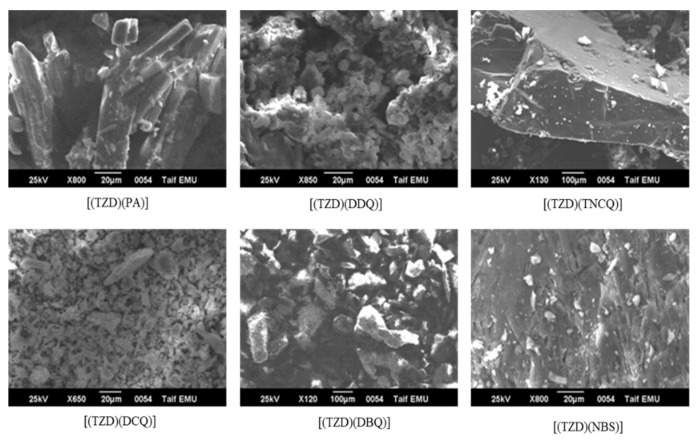
SEM images of the TZD with the six π-acceptors.

Furthermore, XRD and SEM electron microscopy were used to examine the microstructure and morphology of the six TZD charge transfer complexes (Figure 7 and Figure 8). It can be seen that TZD is in complex states, with the sic acceptors in the form of rather crowded particles of various sizes at a 1:1 molar ratio (Figure 7). Due to the complexing process, the six charge transfer complexes have an amorphous structure (Figure 8). Except for TZD-NBS, X-ray powder diffraction demonstrated that the amorphous crystal structures are well crystallized (Figure 7). The raw chemical analysis results for TZD complexes contain a slight discrepancy that fits within the experimental error margins.

### 3.2. Molecular Docking Investigation

The six CT complexes were docked against serotonin (PDB ID: 6BQH). The TZD alone was used as the control for comparative purposes. The potential binding energy of all CT complexes was higher than TZD alone toward the serotonin (Table 1). [(TZD)(DCQ)] had the highest docking energy values of the six CT complexes examined, surpassing the TZD alone. [(TZD)(DCQ)] has theoretical binding energy of −8.9 kcal/mol toward serotonin. On other hand, [(TZD)(DCQ)] has a greater binding energy value towards serotonin than TZD alone, indicating a stronger connection. The docking data of [(TZD)(DCQ)]-serotonin (TD) are displayed in Table 2.

**Figure 9:**
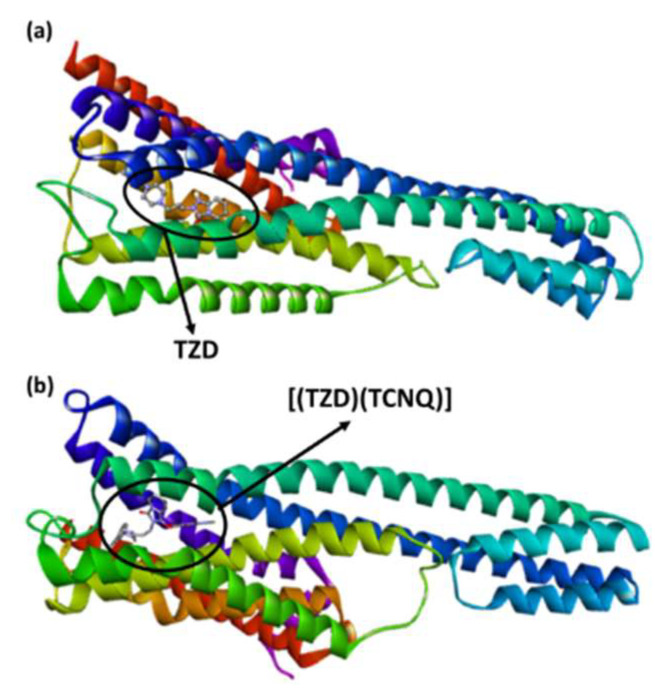
Best docked pose model of serotonin with (**a**): TZD or (**b**): [(TZD)(DCQ)].

Figure 9 shows the molecular docking for the interactions of ligands and receptors. The best docking pose of (TZD)-serotonin as shown in Figure 9b reveals interactions with Val366, Trp367, Val156 (π-Alkyl); Trp336, Phe340 (π-π T-shaped); and Trp151 (π-Sigma).

**Figure 10:**
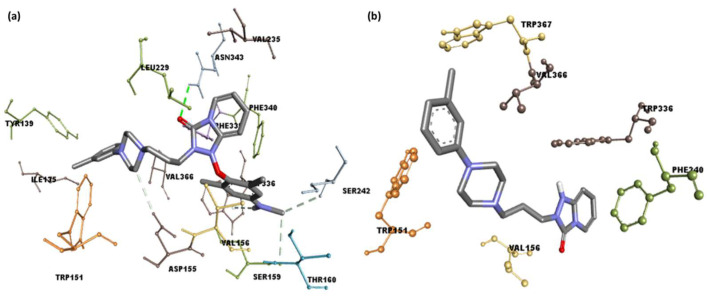
3D structure of interactions between serotonin docked with (**a**) [(TZD)(DCQ)] or (**b**) TZD drug alone.

3D representations of TZD-serotonin or [(TZD)(DCQ)-serotonin are shown in Figure 10.

**Figure 11:**
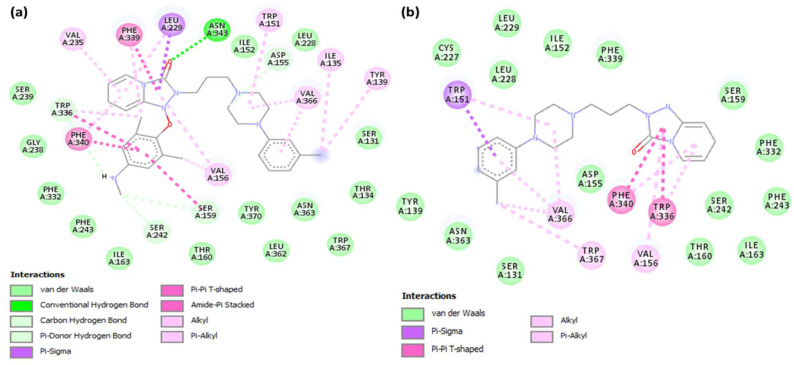
2D structure of the interactions between serotonin with (**a**) [(TZD)(DCQ)] or (**b**) TZD alone.

The hydrogen bond on the [(TZD)(DCQ)]-serotonin complex was established by Asn343 (Figure 10a). There are also interactions between Trp151, Val235, Val156, Val366, Ile135, Typ139 (π-Alkyl); Phe339, Phe340 (π-π T-shaped); and Leu229 (π-Sigma) [45,46]. On the other hand, the theoretical binding energy of the TZD alone with serotonin receptors was −6.5 kcal/mol (Table 1). These findings indicate that the [(TZD)(DCQ)] binds to serotonin more effectively than the reactant donor (TZD drug). The binding energy of [(TZD)(DCQ)]-serotonin is the highest of the six CT complexes. Figure 11 demonstrates 2D depictions of ligand–receptor interactions. Tables S1 and S2 list other details (name, distance, category, and type) of interactions.

**Figure 12:**
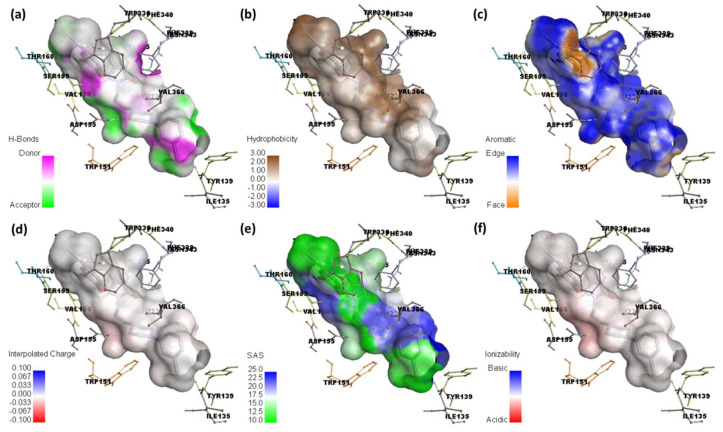
Molecular docking analysis (**a**) hydrogen binding surface, (**b**) hydrophobic surface, (**c**) aromatic surface, (**d**) interpolated charge, (**e**) solvent accessible surface, and (**f**) ionizability surface; between serotonin and [(TZD)(DCQ)] complex.

**Figure 13:**
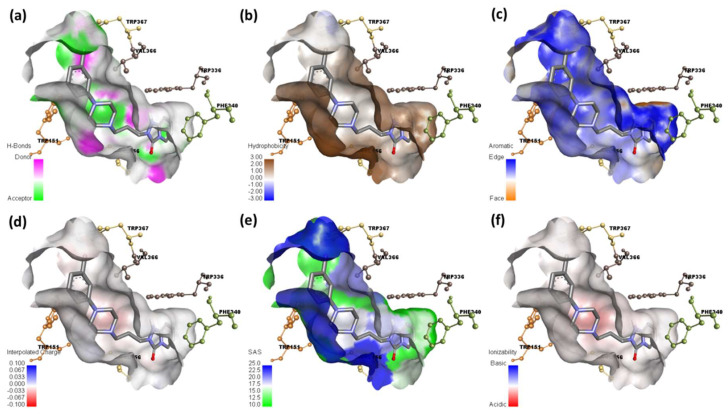
Molecular docking analysis. (**a**) hydrogen binding surface, (**b**) hydrophobic surface, (**c**) aromatic surface, (**d**) interpolated charge, (**e**) solvent accessible surface area, and (**f**) ionizability surface; between serotonin and TZD drug only.

The docked complexes were examined using Discovery Studio software to see what surfaces were near the ligand [47]. Several data of the molecular docking investigation of [(TZD)(DCQ)]-serotonin or (TZD)-serotonin are shown in Figure 12 and Figure 13, respectively.

As illustrated in Figure 12a and Figure 13a, the hydrogen atom acceptor area is green, while the donor area is pink. The hydrophobicity surface confirms the presence of receptor hydrophilicity features around the ligand (Figure 12b and Figure 13b). Using the docking outputs, the aromatic face/edge surface (Figure 12c and Figure 13c, orange/blue = face/edge) has also been illustrated.

The surface area of a receptor that is reachable by a solvent is known as the solvent-accessible surface area (SASA) [48]. Green indicates inadequate accessibility, while blue indicates great accessibility, particularly in the polar zone (Figure 12e and Figure 13e). The acidic and basic propensities were reflected on the ionization surface (Figure 12f and Figure 13f, blue color = basic, red color = acidic) [48].

### 3.3. Structural Stability Analysis upon Ligand Binding

**Figure 14:**
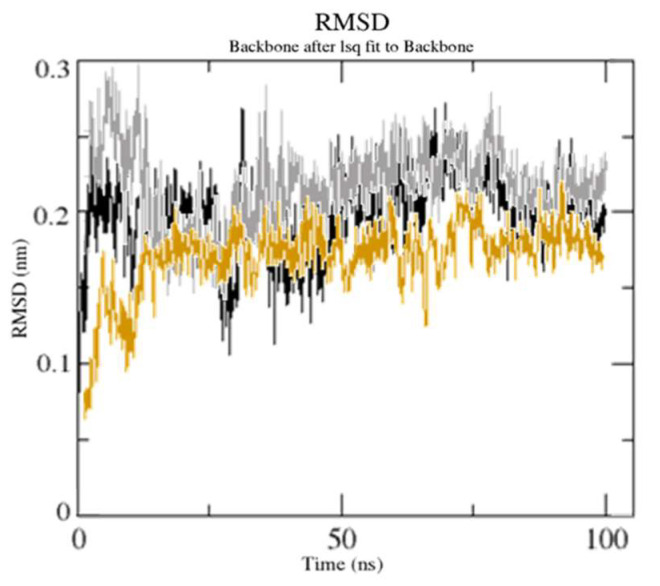
RMSD during 100 ns [unbound serotonin receptor (black), (TZD)-serotonin complex (gray), and [(TZD)(DCQ)]-serotonin complex (orange)].

MD simulation data are handled by computing the RMSD to examine structural stability. After 40 and 30 ns, respectively, both [(TZD)(DCQ)]-serotonin or (TZD)-serotonin acquired stable conformation, with RMSD values of 2.25 Å and 1.65Å (Figure 14).

**Figure 15:**
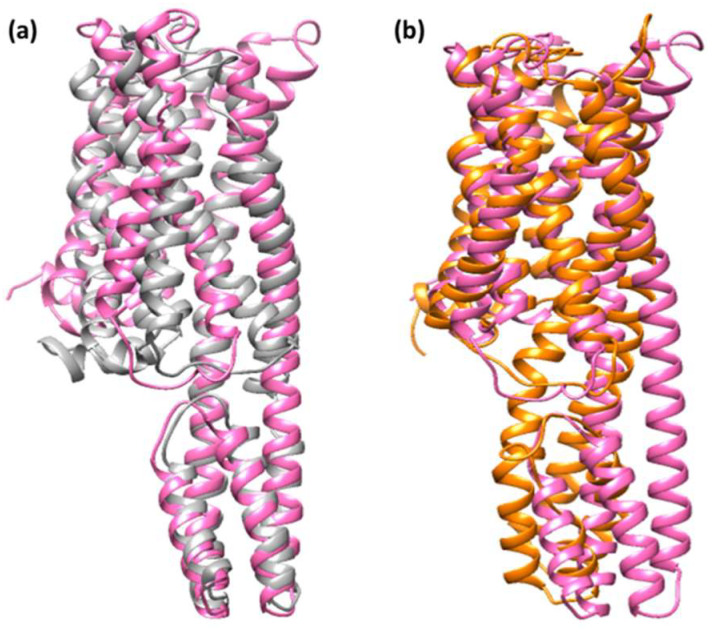
Superimposed structure after simulation of (**a**) (TZD)-serotonin (**b**) [(TZD)(DCQ)]-serotonin. The unbounded serotonin receptor alone showed in pink color, while the serotonin receptor after simulation for (TZD)-serotonin showed in gray and for [(TZD)(DCQ)]-serotonin showed in orange color.

As previously stated, an RMSD value of 3.0 is the most acceptable [49]. Because of ligand (DCQ) binding, the RMSD value for [(TZD)(DCQ)]-serotonin complex has decreased (1.85 Å). This result indicates that [(TZD)(DCQ)]-serotonin creates a more stable combination. As demonstrated in Figure 15, ligand–receptor contact brings protein chains closer together and narrows the distance between them [50].

### 3.4. DFT Investigation

**Figure 16:**
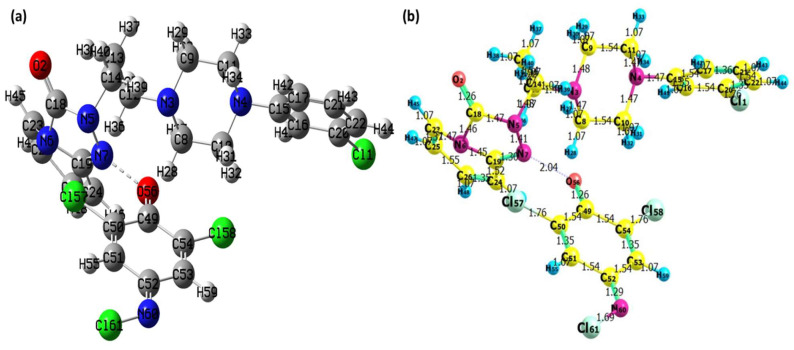
Optimized structure of the [(TZD)(DCQ)] complex; (**a**) Mulliken atom numbering scheme and (**b**) bond lengths.

The [(TZD)(DCQ)] was optimized through the B3LYP/6-311G++ level of theory. The minimum SCF energy after 28 optimization steps was found to be −2165.654571 a.u. Figure 16a shows the optimized shape of the [(TZD)(DCQ)] complex with atomic coordinates and strain-free lattice constants. The bond lengths, as well as the optimized structure of [(TZD)(DCQ)], were achieved and are shown in Figure 16b. Tables S3 and S4 contain information on bond lengths and angles.

**Figure 17:**
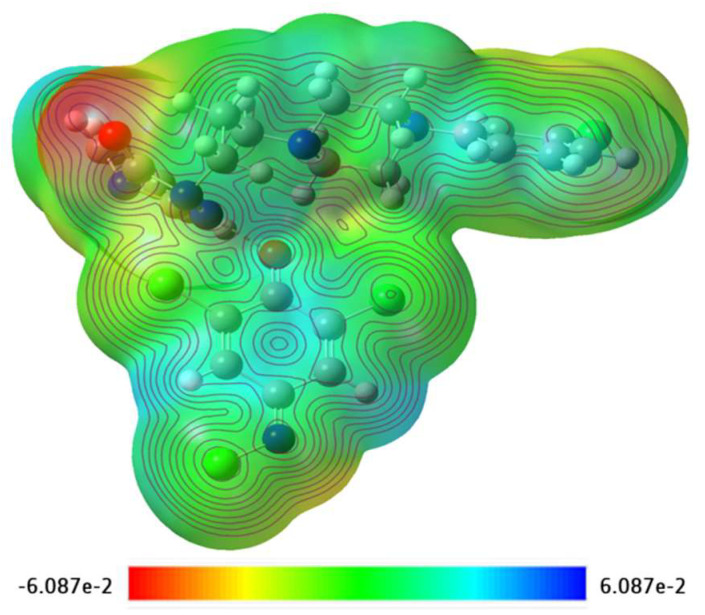
MEP surface map of the CT complex [(TZD)(DCQ)] with respective color scales.

Mulliken charges for [(TZD)(DCQ)] have also been calculated and are shown in Table S5. The MEP map in Figure 17 represents the strength of electrostatic potentials of [(TZD)(DCQ). The electropositive area is shown in blue, whereas the electronegative is shown in red. These findings reveal that electrophilic and nucleophilic assaults prefer to bind to specific locations on the molecule [51]. As illustrated in Figure 17, the MEP surface is mapped using a color scale ranging from −6.087e^−2^ = deep red to +6.087e^−2^ = deep blue [52].

Previously, we found the absorption spectra of [(TZD)(DCQ)] exhibited λmax at 338 nm [27]. TD-DFT in the gas phase was used to investigate the nature of the electronic transitions, and two absorption bands were observed at 310 and 344 nm. The experimental absorption band can be described as a mixture of two absorption transitions at 310 and 344 nm with an average value of 327 nm.

The computed bands at 310 and 344 nm were designated to HOMO-1 → LUMO and HOMO → LUMO, respectively. HOMO are mainly electron donors, which can be seen in the TZD moiety of [(TZD)(DCQ)] complex. While LUMO are electron acceptors, which can be seen in the DCQ moiety of [(TZD)(DCQ)] complex.

**Figure 18:**
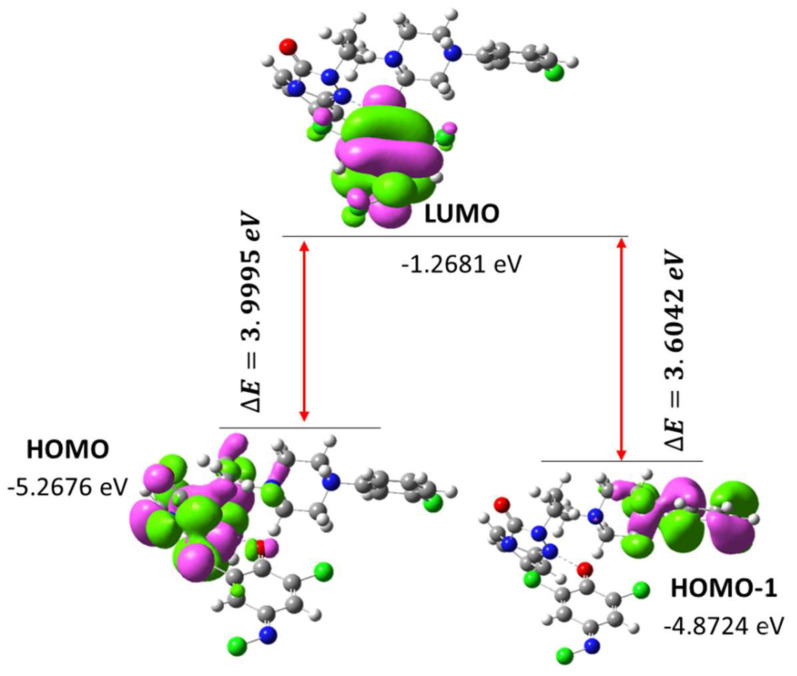
HOMO and LUMO of [(TZD)(DCQ)] complex and their energy gap.

Compounds with a narrower energy gap that is soft in nature have low kinetic stability and higher chemical reactivity [53]. Figure 18 shows the spatial arrangements and HOMO-LUMO gap, as well as associated energies, while Figure S1 describes the MO diagram. For [(TZD)(DCQ)], the HOMO–LUMO and HOMO-1–LUMO gaps (∆E) were found to be 3.9995 and 3.6042 eV, respectively.

Based on the optimized structure and HOMO-LUMO, Table 3 shows several molecular characteristics relevant to chemical reactivity in the gas phase.

Many different drug discovery programs have effectively combined a range of molecular modeling techniques into pharmaceutical research to examine intricate biological and chemical processes [54]. Combining computational and experimental approaches has been incredibly helpful in the discovery and development of novel molecules. The methods of molecular docking, widely utilized in modern drug design, examine the conformations of the ligands, macromolecular targets’ binding sites, where it was adopted. As reported in several published studies, molecular docking has been able to find promising molecules that could one day serve as solutions in crucial areas of human health [55,56,57,58].

## 4. Conclusions

In solid and liquid states, the 1:1 colored TZD and six π-acceptor complexes were evaluated. Several spectroscopic analyses were used to characterize the isolated complexes. The [(TZD)(DCQ)] complex interacts with serotonin receptors more effectively than reactant donor TZD alone, and [(TZD)(DCQ)]-serotonin has the highest binding energy value of all π-acceptor complexes. The [(TZD)(DCQ)] complex is more stable in interaction with the serotonin receptor than TZD, according to a 100-ns MD simulation. The molecular geometry of the [(TZD)(DCQ)]-serotonin complex was investigated using theoretical data acquired from DFT simulations.

## Figures and Tables

**Table 1:** The docking score of TZD alone and TZD with the six synthesized CT complexes against serotonin (PDB ID: 6BQH).

Ligand	Binding Free Energy (kcal/mol)
[(TZD-PA)]	−8.7
[(TZD-NBS)]	−8.3
[(TZD-DDB)]	−8.2
[(TZD–DCQ)]	−8.9
[(TZD-DBQ)]	−7.2
[(TZD–TCNQ)]	−7.4
TZD	−6.5

**Table 2:** Interactions data of TZD–DCQ or TZD alone against serotonin (PDB ID: 6BQH).

Ligand	Binding Free Energy (kcal/mol)	Interactions	
		H-Bond	Others
TZD–DCQ	−8.9	Asn343	Trp151, Val235, Val156, Val366, Ile135, Typ139 (π-Alkyl); Phe339, Phe340 (π-π T-shaped); Leu229 (π-Sigma)
TZD	−6.5		Val366, Trp367, Val156 (π-Alkyl); Trp336, Phe340 (π-π T-shaped); Trp151 (π-Sigma)

**Table 3:** The theoretical molecular characteristics of the [(TZD)(DCQ)] complex.

Parameters	RB3LYP/6-311G++
Minimum SCF energy (a.u.)	−2165.654571
Polarizability (α) (a.u.)	681.95124751
Dipole Moment (Debye)	8.124223274
Zero-point vibrational energy (kcal/mol)	241.2323514
Total thermal energy (kcal/mol)	198.1235462
Electronic spatial extent (a.u.)	40,012.12321
Frontier MO energies (eV)	
LUMO	−1.2681
HOMO	−5.2676
HOMO-1	−4.8724
Gap (HOMO–LUMO)	3.9995
Gap (HOMO-1–LUMO)	3.6042

## Data Availability

Data is contained within article.

## Supplementary Materials

The following supporting information can be downloaded at: https://www.mdpi.com/article/10.3390/ijerph191811281/s1, Table S1. [(TZD)(DCQ]-serotonin interactions results by DS. Table S2. TZD-serotonin interactions result from DS. Table S3. The bond lengths of [(TZD)(DCQ)] were obtained through DFT. Table S4. The bond angles of the [(TZD)(DCQ)] were obtained through DFT. Table S5. Mulliken atomic charges of the [(TZD)(DCQ)] atoms. Figure S1. MO energy level diagram of the [(TZD)(DCQ)] complex.
