# Supplementary material for: Attempting to Increase the Effectiveness of the Antidepressant Trazodone Hydrochloride Drug Using π-Acceptors
Source: Int J Environ Res Public Health. 2022 Sep 8;19(18):11281. doi: 10.3390/ijerph191811281 (PMC9517268; doi:10.3390/ijerph191811281)
Supplement: Supplementary file 1 [file ijerph-19-11281-s001.zip › ijerph-1826076-supplementary.pdf]

Table S1. [(TZD)(DCQ]-serotonin interactions results by DS.

Name	Distance	Category	Type
ASN343:HD21 - [(TZD)(DCQ]:O	2.88554	Hydrogen Bond	Conventional Hydrogen Bond
[(TZD)(DCQ]:C - ASP155:OD2	3.31333	Hydrogen Bond	Carbon Hydrogen Bond
[(TZD)(DCQ]:CL - SER159:O	3.69785	Hydrogen Bond	Carbon Hydrogen Bond
[(TZD)(DCQ]:CL - SER242:O	3.25597	Hydrogen Bond	Carbon Hydrogen Bond
[(TZD)(DCQ]:HN - TRP336	3.13951	Hydrogen Bond	Pi-Donor Hydrogen Bond
TRP336 - [(TZD)(DCQ]	4.87891	Hydrophobic	Pi-Pi T-shaped
PHE340 - [(TZD)(DCQ]	4.72846	Hydrophobic	Pi-Pi T-shaped Pi-
[(TZD)(DCQ] - PHE339	4.76266	Hydrophobic	Pi T-shaped
SER159:C,O;THR160:N - [(TZD)(DCQ]	5.28218	Hydrophobic	Amide-Pi Stacked
LEU229 - [(TZD)(DCQ]	5.08258	Hydrophobic	Alkyl
VAL235 - [(TZD)(DCQ]	4.95457	Hydrophobic	Alkyl
VAL366 - [(TZD)(DCQ]	5.15107	Hydrophobic	Alkyl
[(TZD)(DCQ]:CL - ILE135	4.65913	Hydrophobic	Alkyl
[(TZD)(DCQ]:CL - VAL156	3.80768	Hydrophobic	Alkyl
TYR139 - [(TZD)(DCQ]:CL	4.7308	Hydrophobic	Pi-Alkyl
TRP151 - [(TZD)(DCQ]	4.57405	Hydrophobic	Pi-Alkyl
TRP336 - [(TZD)(DCQ]:CL	3.79169	Hydrophobic	Pi-Alkyl
PHE339 - [(TZD)(DCQ]:CL	4.38122	Hydrophobic	Pi-Alkyl
PHE340 - [(TZD)(DCQ]	5.19771	Hydrophobic	Pi-Alkyl
[(TZD)(DCQ] - VAL156	5.49878	Hydrophobic	Pi-Alkyl
[(TZD)(DCQ] - LEU229	4.7862	Hydrophobic	Pi-Alkyl
[(TZD)(DCQ] - VAL366	4.23249	Hydrophobic	Pi-Alkyl

Table S2. TZD-serotonin interactions results by DS.

Name	Distance	Category	Type
TRP151:CH2 - [(TZD)(DCQ]	3.91922	Hydrophobic	Pi-Sigma
TRP151 - [(TZD)(DCQ]	5.26375	Hydrophobic	Pi-Pi T-shaped
[(TZD)(DCQ] - TRP336	4.8628	Hydrophobic	Pi-Pi T-shaped
[(TZD)(DCQ] - PHE340	4.6715	Hydrophobic	Pi-Pi T-shaped
VAL366 -[(TZD)(DCQ]	5.09644	Hydrophobic	Alkyl
[(TZD)(DCQ]:Cl - VAL366	4.04828	Hydrophobic	Alkyl
TRP151 - [(TZD)(DCQ]	5.26133	Hydrophobic	Pi-Alkyl
TRP336 - [(TZD)(DCQ]	4.77987	Hydrophobic	Pi-Alkyl
TRP336 - [(TZD)(DCQ]	4.78786	Hydrophobic	Pi-Alkyl
PHE340 - [(TZD)(DCQ]	4.80985	Hydrophobic	Pi-Alkyl
TRP367 - [(TZD)(DCQ]:Cl	4.70609	Hydrophobic	Pi-Alkyl
[(TZD)(DCQ] - VAL366	4.79115	Hydrophobic	Pi-Alkyl
[(TZD)(DCQ] - VAL156	5.41572	Hydrophobic	Pi-Alkyl

Table S3. The bond lengths of [(TZD)(DCQ)] obtained through DFT.

S. No.	[(TZD)(DCQ)] (RB3LYP/ 6-311G++)			
	Atom No.	Bond length (Å)	Atom No.	Bond length (Å)
**1**	R(1-20)	1.761	R(15-16)	1.353
**2**	R(2-18)	1.259	R(15-17)	1.54
**3**	R(3-8)	1.47	R(16-20)	1.537
**4**	R(3-9)	1.477	R(16-41)	1.071
**5**	R(3-12)	1.468	R(17-21)	1.357
**6**	R(4-10)	1.469	R(17-42)	1.071
**7**	R(4-11)	1.469	R(19-24)	1.524
**8**	R(4-15)	1.466	R(20-22)	1.354
**9**	R(5-7)	1.411	R(21-22)	1.542
**10**	R(5-14)	1.476	R(21-43)	1.069
**11**	R(5-18)	1.473	R(22-44)	1.071
**12**	R(6-18)	1.455	R(23-25)	1.374
**13**	R(6-19)	1.455	R(23-45)	1.07
**14**	R(6-23)	1.475	R(24-26)	1.352
**15**	R(7-19)	1.297	R(24-46)	1.07
**16**	R(8-10)	1.539	R(25-26)	1.554
**17**	R(8-27)	1.07	R(25-47)	1.068
**18**	R(8-28)	1.069	R(26-48)	1.073
**19**	R(9-11)	1.54	R(49-50)	1.543
**20**	R(9-29)	1.069	R(49-54)	1.537
**21**	R(9-30)	1.071	R(49-56)	1.256
**22**	R(10-31)	1.07	R(50-51)	1.355
**23**	R(10-32)	1.067	R(50-57)	1.759
**24**	R(11-33)	1.071	R(51-52)	1.539
**25**	R(11-34)	1.07	R(51-55)	1.071
**26**	R(12-13)	1.537	R(52-53)	1.542
**27**	R(12-35)	1.071	R(52-60)	1.292
**28**	R(12-36)	1.072	R(53-54)	1.354
**29**	R(13-14)	1.54	R(53-59)	1.071
**30**	R(13-37)	1.072	R(54-58)	1.762
**31**	R(13-38)	1.069	R(60-61)	1.69
**32**	R(14-39)	1.069	R(7-56)	2.038
**33**	R(14-40)	1.069		

Table S4. The bond angles of the [(TZD)(DCQ)] obtained through DFT.

S. No.	[(TZD)(DCQ)] (RB3LYP/ 6-311G++)			
	Atom No.	Bond Angle (Å)	Atom No.	Bond Angle (Å)
**1**	A(1-20-16)	119.8	A(13-12-35)	109.1
**2**	A(1-20-22)	120.3	A(13-12-36)	108.9
**3**	A(2-18-5)	125.4	A(12-13-14)	110.2
**4**	A(2-18-6)	125.8	A(12-13-37)	109.5

**5**	A(8-3-9)	110.2	A(12-13-38)	109
**6**	A(8-3-12)	108.6	A(35-12-36)	109.7
**7**	A(3-8-10)	109.8	A(14-13-37)	110.4
**8**	A(3-8-27)	109.5	A(14-13-38)	108.1
**9**	A(3-8-28)	109.4	A(13-14-39)	110.9
**10**	A(9-3-12)	108.4	A(13-14-40)	109.2
**11**	A(3-9-11)	110.2	A(37-13-38)	109.5
**12**	A(3-9-29)	109.5	A(39-14-40)	109.3
**13**	A(3-9-30)	109.1	A(16-15-17)	120
**14**	A(3-12-13)	110.7	A(15-16-20)	120.2
**15**	A(3-12-35)	108.4	A(15-16-41)	119.8
**16**	A(3-12-36)	110.1	A(15-17-21)	119.9
**17**	A(10-4-11)	108.9	A(15-17-42)	120.1
**18**	A(10-4-15)	109.7	A(20-16-41)	120
**19**	A(4-10-8)	109.1	A(16-20-22)	119.9
**20**	A(4-10-31)	109.6	A(21-17-42)	120
**21**	A(4-10-32)	109.4	A(17-21-22)	119.9
**22**	A(11-4-15)	110.3	A(17-21-43)	120.1
**23**	A(4-11-9)	109.1	A(19-24-26)	117.3
**24**	A(4-11-33)	109.7	A(19-24-46)	121.4
**25**	A(4-11-34)	109.5	A(20-22-21)	120.1
**26**	A(4-15-16)	118.9	A(20-22-44)	120
**27**	A(4-15-17)	121.1	A(22-21-43)	120
**28**	A(7-5-14)	113	A(21-22-44)	120
**29**	A(7-5-18)	102.4	A(25-23-45)	120.1
**30**	A(5-7-19)	111.7	A(23-25-26)	121
**31**	A(5-7-56)	123.9	A(23-25-47)	119.6
**32**	A(14-5-18)	105.6	A(26-24-46)	121.3
**33**	A(5-14-13)	106.9	A(24-26-25)	119
**34**	A(5-14-39)	110.6	A(24-26-48)	120.1
**35**	A(5-14-40)	109.9	A(26-25-47)	119.5
**36**	A(5-18-6)	108.8	A(25-26-48)	120.9
**37**	A(18-6-19)	99.4	A(50-49-54)	120.1
**38**	A(18-6-23)	116.7	A(50-49-56)	119.9
**39**	A(19-6-23)	112.7	A(49-50-51)	120.1
**40**	A(6-19-7)	111.8	A(49-50-57)	119.9
**41**	A(6-19-24)	121.7	A(54-49-56)	120
**42**	A(6-23-25)	120.2	A(49-54-53)	119.9
**43**	A(6-23-45)	119.8	A(49-54-58)	119.8
**44**	A(7-19-24)	126.5	A(49-56-7)	119.6
**45**	A(19-7-56)	124.4	A(51-50-57)	120
**46**	A(10-8-27)	109.1	A(50-51-52)	119.6
**47**	A(10-8-28)	109.5	A(50-51-55)	120.4
**48**	A(8-10-31)	110	A(52-51-55)	120
**49**	A(8-10-32)	109.3	A(51-52-53)	120.4
**50**	A(27-8-28)	109.5	A(51-52-60)	119.4

**51**	A(11-9-29)	109.5	A(53-52-60)	120.2
**52**	A(11-9-30)	108.9	A(52-53-54)	119.9
**53**	A(9-11-33)	109.2	A(52-53-59)	120.2
**54**	A(9-11-34)	109.9	A(52-60-61)	119.9
**55**	A(29-9-30)	109.5	A(54-53-59)	119.9
**56**	A(31-10-32)	109.5	A(53-54-58)	120.2
**57**	A(33-11-34)	109.4		

Table S5. Mulliken atomic charges of the [(TZD)(DCQ)] atoms.

**S. No.**	**Synthesized complex**			
	**Mulliken atomic numbers**	**Mulliken atomic charges**	**Mulliken atomic numbers**	**Mulliken atomic charges**
**1**	1Cl	-0.04942	32H	0.16865
**2**	2O	-0.54516	33H	0.17958
**3**	3N	-0.54281	34H	0.16717
**4**	4N	-0.66525	35H	0.13573
**5**	5N	-0.46077	36H	0.31159
**6**	6N	-0.6965	37H	0.17188
**7**	7N	-0.3286	38H	0.22389
**8**	8C	-0.13684	39H	0.22325
**9**	9C	-0.13969	40H	0.21835
**10**	10C	-0.20156	41H	0.19948
**11**	11C	-0.16839	42H	0.19277
**12**	12C	-0.13341	43H	0.17103
**13**	13C	-0.50297	44H	0.19276
**14**	14C	-0.09915	45H	0.2377
**15**	15C	0.25538	46H	0.21345
**16**	16C	-0.08244	47H	0.19118
**17**	17C	-0.13231	48H	0.1895
**18**	18C	0.78762	49C	0.59717
**19**	19C	0.40068	50C	-0.30143
**20**	20C	-0.26758	51C	-0.0586
**21**	21C	-0.1559	52C	0.2488
**22**	22C	-0.0655	53C	0.0574
**23**	23C	0.09151	54C	-0.35593
**24**	24C	-0.15677	55H	0.27504
**25**	25C	-0.21765	56O	-0.40821
**26**	26C	-0.11492	57Cl	0.1072
**27**	27H	0.1532	58Cl	0.04778
**28**	28H	0.19075	59H	0.24535
**29**	29H	0.17018	60N	-0.56297
**30**	30H	0.16677	61Cl	0.1523
**31**	31H	0.21561		

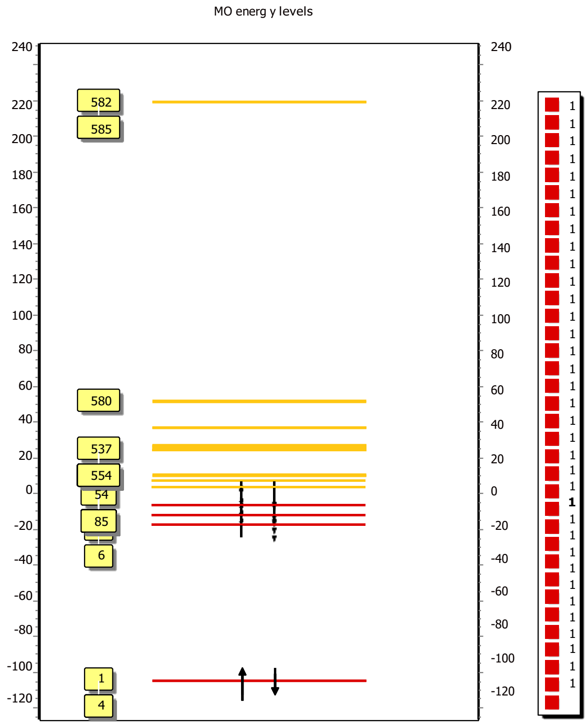

Figure S1. MO energy level diagram of the [(TZD)(DCQ)] complex.
